# Modification of corn starch by thermal-ultrasound treatment in presence of Arabic gum

**DOI:** 10.1038/s41598-022-23836-z

**Published:** 2022-11-11

**Authors:** Abdolkhalegh Golkar, Jafar Mohammadzadeh Milani, Ali Motamedzadegan, Reza Esmaeilzadeh Kenari

**Affiliations:** Department of Food Science and Technology, Sari Agricultural Sciences and Natural Resources University, Sari, Iran

**Keywords:** Carbohydrates, Chemical modification

## Abstract

This research extends the effects of a thermal-ultrasound treatment (at 25, 45, and 65 °C for 30 and 60 min) on the physicochemical, structural, and pasting properties of corn starch in presence of Arabic gum. Treated samples had lower leached amylose compared with corn starch, but it was non-significant (*p* < 0.05). In comparison to alone corn starch and a combination of Arabic gum, thermal-ultrasound treatment increased the swelling power and solubility of samples. Treatment significantly (*p* < 0.05) decreased the syneresis of treated starch gels, especially at a temperature of < 45 °C, but paste clarity was increased at the higher temperature (65 °C). The enthalpy of treated samples was in the range 15.20–16.37 J/g. Sonication at 65 °C for 60 min had the most destructive effect on corn starch granules, but at 30 min granules were swollen only. FT-IR spectra of samples confirmed the physical modification of thermal-ultrasound treatment. The relative crystallinity index of samples changed in the range 21.88–35.42% and decreased with rising time and temperature. Sonication at 45 °C for 30 and 60 min produced starch-gum mixtures with different pasting properties. Thermal-ultrasound treatment in presence of gum can be a viable technique to modify starches with different functionality.

## Introduction

Starch is an abundant, low price, and natural hydrocolloid that possesses wide applications in food science; but due to the drawbacks of native starch causes limited use in some food applications^1^. Native starch is usually modified chemically, enzymatically, genetically, and physically to develop starch-based food products. Nowadays, physical modifications are gaining importance in food industries due to the limited use of chemical agents, less processing time, and environment-friendly processing^2^.

Ultrasound is one of the physical methods for the modification of starch. The use of ultrasound to modify the starch's functionality has increased in the last decades^3,4^. Ultrasonication locally creates strong shear force, high temperature, and free radicals which change the structure and properties of starch. The extent of changes depends on the frequency and intensity of ultrasound, time, temperature, and moisture of the system, as well as the starch origin. In addition, ultrasound had been connected to the emerging concept of “green chemistry and technology” for environmentally friendly applications^5^.

Thermal treatment offers the potential to change starch functionality at a low-cost method. Pregelatinized starches are widely used for many foods as a major ingredient to provide thickening texture at temperatures below the gelatinization temperature^6^. When both heat and ultrasound waves act simultaneously, high sensitivity on microbial cell walls occurs, causing damage to the cell structure, as a consequence of the called "additive effect". Thermosonication or thermal-ultrasound treatments are the terms that are usually applied to this technique^7^. In the previous work, we studied the effect of thermal-ultrasound treatment on the physicochemical and rheological properties of corn starch. Results showed that thermal ultrasound is a viable technique for starch modification compared to conventional thermal and ultrasound treatments^4^.

On the other hand, the engineered selection of hydrocolloids in starch-based products could be influenced by the physicochemical and rheological properties of starch. Hydrocolloids are often modified the functional properties of starch to overcome undesirable changes in food products during processing (such as breakdown of viscosity after shearing) and self-life (such as retrogradation) without chemical modification. Usually, if the starch was applied with a small content of hydrocolloids in food formulation, the desirable quality, and textural characteristics may be obtained that do not generally exhibit ideal functional properties for the preparation of food products^8^. Arabic gum is generally used as an emulsifier/or stabilizer in food systems. It is a weak polyelectrolyte that transfers carboxyl groups and can be applied as a dietary fiber. Due to the well-known structure of Arabic gum and the numerous researches about the effect of its in-model systems, this gum was selected.

Today, dual modification of starch has been most attractive, because often the use of a single modification method is not complete and effective to improve the application and processing characteristics of native starches^9^. Therefore, this study aimed to investigate the effect of thermal-ultrasound treatment in the combination of Arabic gum on the amylose content, swelling power and solubility, syneresis, paste clarity, differential scanning calorimetry, Fourier transform infrared spectra (FT-IR), scanning electron microscopy (SEM), X-ray diffraction (XRD), and pasting characteristics of corn starch.

## Results and discussion

### Amylose content

The leached amylose plays an important role in the further gelation and retrogradation of the starch paste. The amount of leached amylose was measured for treated samples, and the results are presented in Table 1.

**Table 1:** Amylose content, syneresis, and paste clarity (after 2 and 7 days) of thermal-ultrasound treated corn starch-Arabic gum mixtures.

Sample	Leached amylose content (%)	Syneresis (%)		Clarity	
		2 days	7 days	2 days	7 days
S-G2530	21.15 ± 1.48^ab^	65.25 ± 0.05^a^	66.95 ± 1.38^a^	2.12 ± 0.01^a^	2.50 ± 0.01^ab^
S-G2560	21.67 ± 0.25^ab^	58.65 ± 1.55^c^	62.75 ± 0.18^ab^	2.15 ± 0.01^a^	2.51 ± 0.01^ab^
S-G4530	23.57 ± 0.74^a^	58.29 ± 1.41^c^	62.38 ± 2.68^b^	2.13 ± 0.01^a^	2.41 ± 0.03^c^
S-G4560	19.57 ± 0.74^b^	60.71 ± 0.45^bc^	61.29 ± 1.50^b^	2.15 ± 0.01^a^	2.46 ± 0.01^b^
S-G6530	22.37 ± 2.72^ab^	62.56 ± 1.47^ab^	62.73 ± 1.32^ab^	1.96 ± 0.15^b^	2.47 ± 0.03^ab^
S-G6560	22.05 ± 1.69^ab^	60.20 ± 2.11^bc^	61.06 ± 0.85^b^	1.96 ± 0.15^b^	2.35 ± 0.07^c^
S-G	22.55 ± 0.00^a^	63.73 ± 0.00^a^	60.45 ± 2.30^b^	2.15 ± 0.01^a^	2.52 ± 0.01^a^
S	22.92 ± 0.25^a^	62.71 ± 0.12^ab^	59.72 ± 2.45^b^	2.11 ± 0.01^ab^	2.50 ± 0.00^ab^

^a–c^For each column, similar small code letters are not significantly different at *p* < 0.05.

It was observed that the amylose content of corn starch was 22.92 ± 0.25%. Primarily, Arabic gum (S-G) slightly decreased the leached amylose from corn starch (S); but this effect was non-significant (*p* > 0.05). Arabic gum may be inhibited the leaching of amylose by the coverage of the granule's surface. This phenomenon was consistent with the previous results reported by Sheng et al.^10^ for tapioca starch-pullulan mixtures and Zhang et al.^11^ for corn starch and Arabic gum systems. Some researchers reported that the amount of leached starch polymer was significantly lower owing to the increased viscosity of the continuous phase by the presence of hydrocolloids, which prevent the diffusion of starch polymer from starch granules^12^. These findings were not confirmed for corn starch and Arabic gum mixtures due to lower viscosity of the gum in comparison to other gums such as xanthan and guar gums.

Results showed that treated samples (except S-G4530) had lower leached amylose compared with corn starch (S); but it was non-significant (*p* < 0.05). Based on previous reports by Chan et al.^13^ and Falsafi et al.^14^, leached amylose increased after sonication which might be due to the amylose and amylopectin chain cleavages. In contrast, in this study, the amylose content of treated samples was decreased in presence of Arabic gum. This reduced trend could be discussed by starch molecules and Arabic gum interactions. Generally, leached amylose increased with increasing temperature from 25 to 65 °C. But the time of ultrasound had an unclear trend. Except for S-G4560, other sonicated samples showed non-significant differences at 0.05 (*p* > 0.05) compared with corn starch (S). The S-G4530 sample had higher leached amylose than the S-G sample. This phenomenon might be related to the cleavage of amylopectin and long amylose chains that increase the mobility of molecules for the reaction with iodine. In the case of S-G4560, it should be noted that starch molecules' interactions may be reduced by spectrophotometric measurement values. It should be noted that the lower leached amylose content can be modified by the other functional properties that require doing other tests.

### Syneresis (%)

The retrogradation behavior of non-treated and thermal-ultrasound-treated samples was measured by studying syneresis (%) during storage at 4 °C for 2 and 7 days (Table 1).

The syneresis of all samples (except S and S-G samples) increased with increasing the time of storage to 7 days. The S and S-G samples showed lower syneresis during storage; but at the first, showed higher values. Ultrasonication significantly (*p* < 0.05) decreased the syneresis of treated gels especially S-G2560 and S-G4530 samples compared with S and S-G. But retrogradation in these samples was observed after 7 days at 4 °C. The retrogradation properties of starches are indirectly influenced by the structural arrangement of starch chains within the amorphous and crystalline regions of the ungelatinized granule, which in turn, influence the extent of granule breakdown during gelatinization and the interactions that occur between starch chains during gel storage^1^. Thus, sonication at 25 °C for 30 min and 45 °C for 60 min and at a higher temperature of 45 °C resulted in increased syneresis and retrogradation tendency. This result may be to the appropriate conditions of corn starch and Arabic gum mixtures (S-G2560 and S-G4530) after sonication that inhibited retrogradation. Because at 25 °C for 30 min, ultrasound was not changed considerably structure of the S-G sample, moreover at 65 °C also due to the decrease in ultrasound effects with increasing temperature occurred similar phenomenon. As the high temperature in a compressed cavitation bubble was not a prerequisite for producing strong shear forces in the vicinity of the bubble. Increasing the reaction temperature allowed cavitation to be achieved at lower acoustic intensity. This was a direct consequence of the rising vapor pressure associated with the liquid heated^15^. At 65 °C, the normal gelatinization of corn starch achieved the leakage of starch molecules to the medium and caused retrogradation during storage. The results imply that the amount of amylose and amylopectin in starch gels is the main factor that plays a vital role in influencing the retrogradation properties. It should be noted that the effect of Arabic gum on the deterioration of starch molecular interactions also has been considered.

Generally, the substitution of part of corn starch (4.75%) by Arabic gum (0.25%) in S-G mixtures (5% total biopolymer concentration) and decreasing the starch content in all samples, caused lower water holding capacity that resulted in increasing syneresis in all S-G mixtures with and without treatment.

### Paste clarity

Paste clarity is one of the most main functional characteristics of starch in food systems. Thus, the absorbance of the starch pastes for 2 and 7 days at 4 °C was measured. The results of the paste clarity of treated samples were reported in Table 1. In all samples, the absorbance was increased during storage i.e., paste clarity decreased. It should be noted that the paste clarity of samples was significant after 7 days, and S-G4530 and S-G6560 showed lower values (absorbance) than other samples.

For the first time, paste clarity increased only at 65 °C for 30 and 60 min (decreasing absorbance). The effect of ultrasound was lower at a temperature lower than 45 °C (*p* > 0.05). Amini et al.^16^ reported that ultrasonication at temperatures below 45 °C decreased slightly the paste clarity; a profound increase was observed at the higher temperatures. They found that the sonicated samples were more transparent than the non-sonicated ones^16^. However, this statement was not in agreement with the result of this study and may be related to the existence of Arabic gum and biopolymer interactions between starch molecules and Arabic gum which can induce a higher level of light absorption. Increased solubility and decreased amount of starch granules (swollen form) resulted in higher clarity. So, the disintegration of corn starch granules at 65 °C may occur. But in some cases, the higher solubility of starch pastes by amylose and amylopectin chains caused the higher retrogradation during storage. Although, the electrostatic interaction of starch molecules with Arabic gum that forms interpolymeric complexes resulted in increased turbidity during storage.

Although, other researchers also reported that the increasing clarity of oat starch paste after an ultrasound resulted in the increased solubility of starch and the decreased amount of starch granules remnants^14^. Krishnakumar and Sajeev^17^ observed the clarity of the cassava pastes prepared using ultrasonicated samples to be lower compared with the control but it was not much statistically different (*p* < 0.05).

### Swelling power and solubility

The swelling power and solubility are the main parameters that provide information on the quantity of biopolymer chain interactions in starch granules due to the amorphous and crystalline regions. The swelling power is well-defined as the capacity of molecules to hold water in the starch network by hydrogen bonds. In general, the rheological properties of starch are significantly affected by the swelling power and solubility of starch^18^. The swelling power and solubility of samples were reported in Table 2. In all samples, these parameters increased with increasing temperature from 25 to 85 °C (*p* < 0.05). The swelling power of thermal-ultrasound-treated samples significantly (*p* < 0.05) increased with increasing time and temperature of ultrasound. S-G6560 showed the highest value (34.02 ± 0.80%) at 85 °C. It has been reported that the increased swelling power is affected by ultrasound due to the breakdown of intermolecular bonds and starch granule aggregates, changes in the crystalline structure, and bonding of water molecules^19–21^.

**Table 2:** Swelling power and solubility of thermal-ultrasound treated samples at 25, 45, 65, and 85 °C.

Sample	25 °C	45 °C	65 °C	85 °C
S-G2530	1.34 ± 0.05^cD^	5.41 ± 0.57^bcC^	13.07 ± 0.73^cdB^	18.24 ± 0.23^deA^
S-G2560	1.33 ± 0.35^cD^	6.14 ± 0.97^bcC^	13.18 ± 0.95^cB^	19.83 ± 0.95^dA^
S-G4530	3.31 ± 0.76^bC^	6.78 ± 0.09^bC^	14.32 ± 0.10^bcB^	23.45 ± 2.90^cA^
S-G4560	2.30 ± 0.63^bcD^	7.20 ± 0.77^bC^	11.64 ± 1.00^cdB^	21.45 ± 0.86^cdA^
S-G6530	7.00 ± 1.43^aC^	10.63 ± 0.45^aC^	18.05 ± 3.26^abB^	29.25 ± 2.67^bA^
S-G6560	8.45 ± 0.42^aC^	11.32 ± 1.60^aC^	22.28 ± 3.95^aB^	34.02 ± 0.80^aA^
S-G	1.41 ± 0.46^cC^	2.95 ± 0.27^dC^	8.65 ± 1.03^ dB^	14.01 ± 0.79^fA^
S	1.19 ± 0.06^cD^	4.92 ± 0.46^cC^	11.62 ± 0.60^cdB^	15.93 ± 0.59^efA^
**Solubility**				
S-G2530	1.16 ± 0.04^cA^	5.21 ± 0.47^bcB^	13.58 ± 0.60^cC^	19.94 ± 0.10^deD^
S-G2560	1.41 ± 0.07^cA^	5.87 ± 0.88^bcB^	13.60 ± 1.01^cC^	21.92 ± 1.24^cdD^
S-G4530	2.90 ± 0.71^bA^	6.53 ± 0.19^bB^	14.66 ± 0.71^bcC^	26.21 ± 3.83^cC^
S-G4560	2.03 ± 0.60^bcA^	6.91 ± 0.66^bB^	12.04 ± 0.95^cdC^	23.85 ± 1.34^cdD^
S-G6530	6.08 ± 1.17^aA^	10.21 ± 0.55^aB^	18.71 ± 3.51^abC^	32.16 ± 2.91^bC^
S-G6560	7.32 ± 0.32^aA^	10.92 ± 1.72^aB^	22.93 ± 4.02^aC^	37.43 ± 0.82^aC^
S-G	1.25 ± 0.34^cA^	2.84 ± 0.30^ dB^	8.90 ± 1.05^dC^	15.48 ± 0.76^fC^
S	1.08 ± 0.13^cA^	4.69 ± 0.44^cB^	12.11 ± 0.80^cdC^	17.57 ± 0.75^efD^

^a-f^For each column, similar small code letters are not significantly different at *p* < 0.05.

^A-D^For each row, similar large code letters are not significantly different at *p* < 0.05.

In the case of solubility, the results showed a similar trend compared to swelling power reports. Ultrasound increased the solubility of samples and its effect was higher at 85 °C than at 25 °C. The solubility of S-G samples at 25 °C was 1.25 ± 0.34% and increased to 15.48 ± 0.76% after heating. This value was 1.41 ± 0.07% for S-G2560, and then significantly increased to 21.92 ± 1.24%. Therefore, thermal-ultrasound-treated corn starch and Arabic gum mixture at 25 °C for 60 min could be increased the solubility of the mixture by approximately 25% higher than one at 85. This effect was increased at the high temperature and time of the ultrasound. For example; S-G6560 showed a solubility of 37.43 ± 0.82% at 85 °C. This value increased by over 200% in comparison to the S-G sample. Although, it should be noted that the solubility of S and S-G samples at higher temperatures were 17.57 ± 0.75 and 15.48 ± 0.76%, respectively.

Amini et al*.*^16^ found that the solubility index of alone corn starch sharply increased (about twofold and more) after ultrasonication at 65 °C, but there was no difference between the solubility of samples measured at 25 and 45 °C. Generally, the increased solubility of samples is attributed to the amylose-associated leakage outside the granules^22^. The amylose content results showed no significant between thermal-ultrasound treated samples and S-G, but the solubility of samples was significant at *p* < 0.05. On the other side, it is reported that the increased surface area of granules due to the pores and channels after ultrasound permits water molecules to diffuse more easily into the granules and results in the solubility increasing^21,23^. In the corn starch and Arabic gum mixtures, the electrostatic interaction of two biopolymers after treatment can influence this property. Therefore, corn starch and Arabic gum mixtures rearranged after thermal-ultrasound treatment and produced a new complex with higher solubility and swelling power properties.

In comparison to S and S-G samples, Arabic gum might be decreased or increased the solubility and swelling power of corn starch. Results showed that these values of samples decreased in presence of Arabic gum which may be related to the substitution of gum with 0.25% of corn starch. Because the water-holding capacity of Arabic gum is lower than corn starch. But in a mixture of corn starch and Arabic gum after the ultrasound, the attracted results were calculated. Although, Arabic gum without treatment decreased the solubility and swelling power of corn starch at the higher temperature (85 °C). But this value was increased after thermal-ultrasound in all samples, especially in S-G6560 after heating at the elevated temperature. The different behavior of swelling power and solubility of S-G4560 sample may be related to the increased interactions involving amylose-amylose and amylose-amylopectin chains.

Based on the ultrasound effect and temperature on corn starch and their effect on the Arabic gum structure and their biopolymer interaction, this treatment could be designed as a corn starch-Arabic gum mixture with higher swelling power.

### Differential scanning calorimetry (DSC)

DSC results of thermal-ultrasound-treated starches are summarized in Table 3. To and Tp of corn starch-Arabic gum sample (S-G) was approximately higher than alone corn starch (S); moreover, enthalpy of S-G was higher than S. In fact, Arabic gum increased enthalpy of S-G system. It seems that Arabic gum might be enwrapped to the corn starch granules, thus limiting the amount of the leached starch molecules.

**Table 3:** Thermal properties, structural characteristics, and relative crystallinity (%) of thermal-ultrasound treated samples.

Sample	Thermal properties			Structural characteristics		Relative crystallinity (%)
	To (°C)	Tp (°C)	ΔH (J/g)	IR ratio 1047/1022	IR ratio 1022/995	
S-G2530	67.5	80.3	17.62	1.119	0.654	33.91
S-G2560	66.9	77.9	18.13	1.133	0.641	32.32
S-G4530	66.0	77.0	16.07	1.075	0.667	32.44
S-G4560	71.3	87.7	16.81	1.073	0.674	31.32
S-G6530	71.7	81.8	16.38	1.111	0.676	26.55
S-G6560	72.3	79.2	15.20	1.035	0.671	21.88
S-G	68.4	84.5	18.77	1.182	0.625	33.39
S	66.4	82.6	16.37	1.157	0.646	35.42

Each value is the average of three independent experiments.

In the case of the treated sample, To, Tp, and ΔH were changed at 66.0–72.3 °C, 77.0–87.7 °C, and 15.20–18.13 J/g, respectively. Generally, To and Tp were increased, but ΔH decreased after thermal-ultrasound treatment, especially at the higher temperature (65 °C). The higher To, Tp, and ΔH values were related to the S-G6560, S-G4660, and S-G2560 samples, respectively. It may be concluded that ultrasound disorganized the structure of corn starch granules, but Arabic gum delayed the gelatinization by the coverage of the granule's surface. The results showed that the onset and peak temperatures were slightly shifted towards higher values for sonicated samples compared with control, except for the sample sonicated at 65 °C. But the sample sonicated at 65 °C had lower ΔH values compared with the non-sonicated one. The organization of the amylose and amylopectin components of starch plays an important role in the gelatinization of starches treated by ultrasound.

Ultrasound waves induced a rearrangement of the molecular packing within the granule microstructure. A decrease in the enthalpy of sonicated oat starch was reported by Falsafi et al.^14^.

### Scanning electron microscopy (SEM)

**Figure 1:**
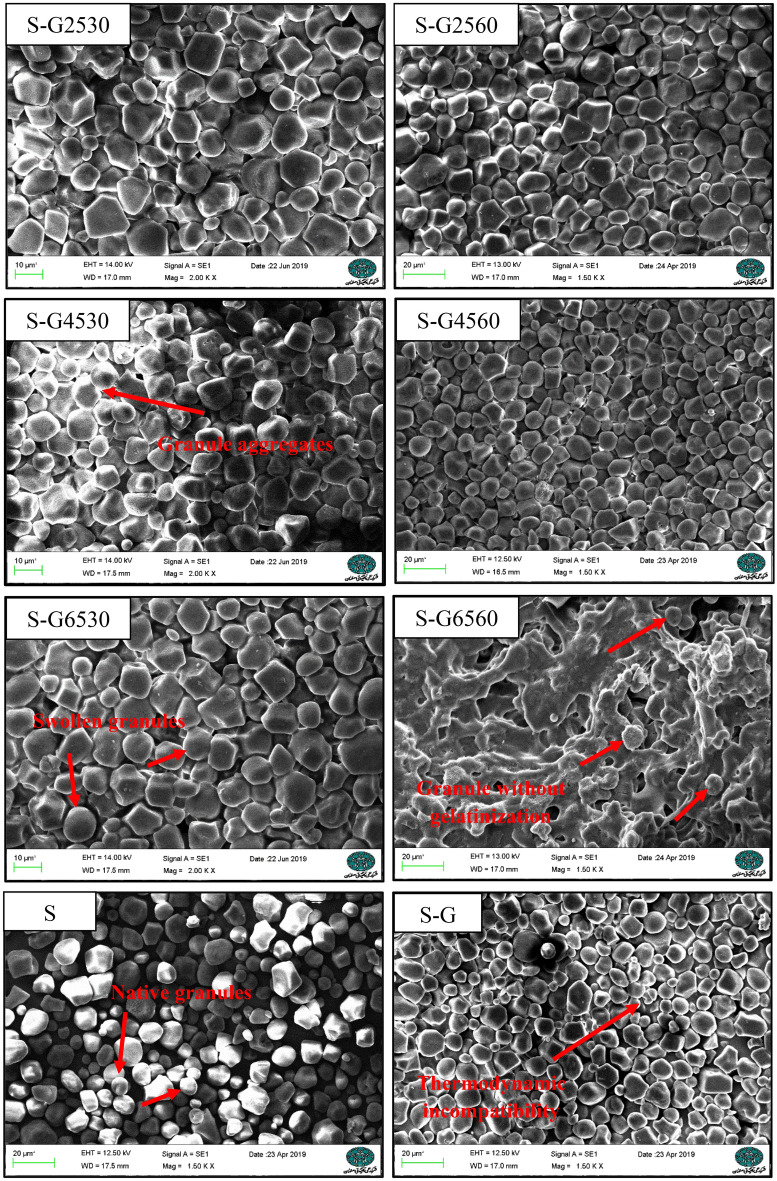
SEM micrographs of thermal-ultrasound treated corn starch-Arabic gum mixtures.

The morphology of treated samples is studied by SEM microscopic observation (Fig. 1). Based on S and S-G micrographs, aggregation occurred before thermal-ultrasound treatment which may be related to the thermodynamic incompatibility (associative or segregative) between corn starch and Arabic gum. In micrographs at a temperature lower than 45 °C, all starch granules remained unchanged. Moreover, due to the leakage of amylose from corn starch, the aggregation of granules has been revealed.

At the higher temperature (65) for 30 min, starch granules were swollen, but at the longer time (60 min) approximately all of the granules changed and formed an amorphous matrix with some of the unchanged granules. In the case of S-G6560, it can be observed that the wrapping effect of Arabic gum on the cornstarch granules inhibited starch gelatinization. Although the temperature of ultrasound treatment (65 °C) is lower than the gelatinization temperature of corn starch, a combination of ultrasound and thermal treatment could promote the gelatinization phenomenon, resulting in increased gelatinization. Ultrasonication caused severe physical damage on the granular surface, by creating fissures or cracks, thereby increasing the ability of the granules to retain more water^4^.

### Fourier transform infrared (FT-IR) spectra

**Figure 2:**
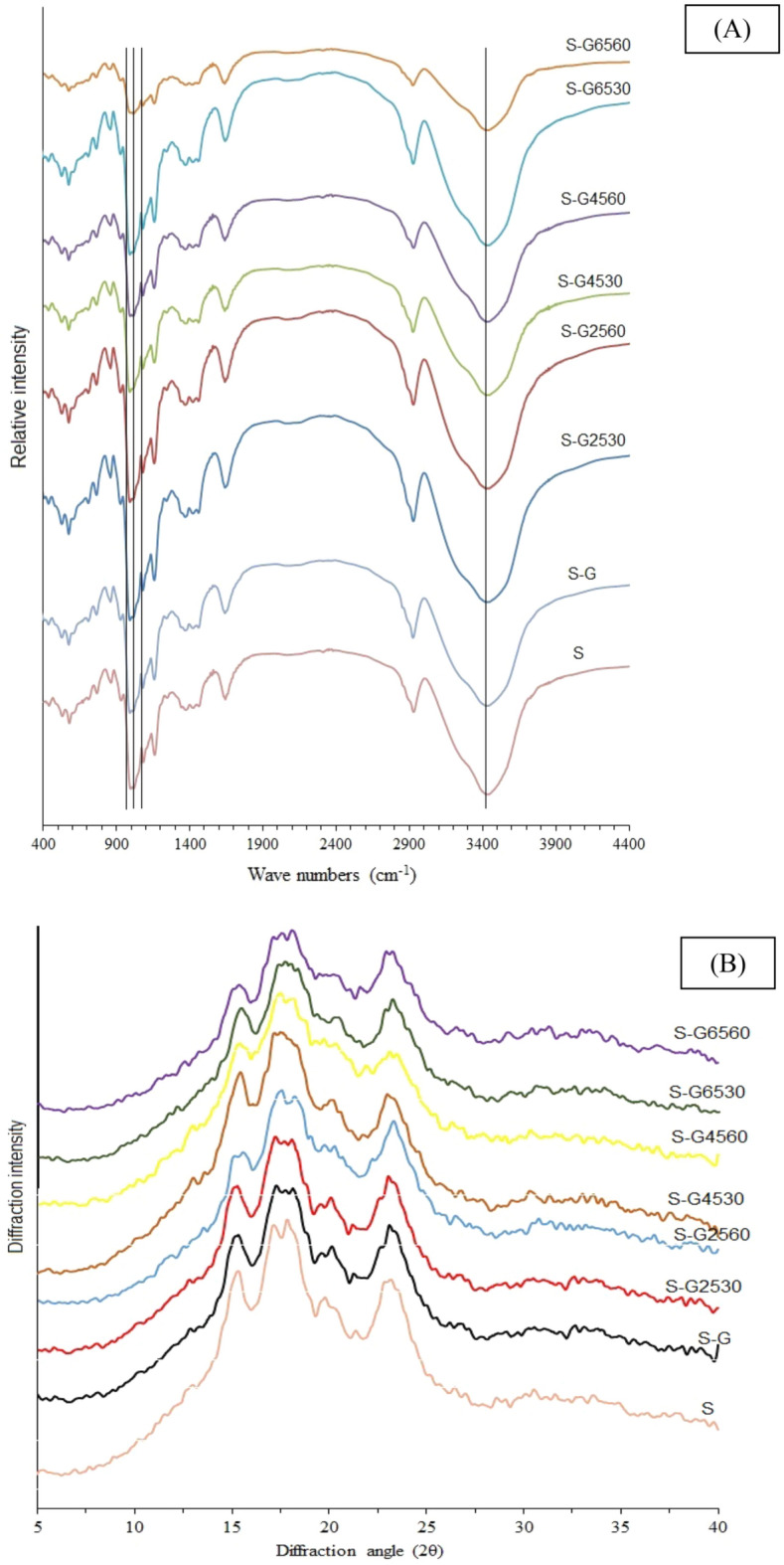
(**A**) FT-IR spectra and (**B**) XRD diffractogram of thermal-ultrasound treated corn starch-Arabic gum mixtures.

The FT-IR spectra of thermal-ultrasound-treated samples are compared in Fig. 2. The intensity of the peak altered after ultrasonication with Arabic gum. In some samples intensity of peaks increased after treatment; but in some cases, such as S-G6560, the intensity was decreased. There was an increase in interactions between corn starch and Arabic gum molecules and more hydrogen bonds were formed after the ultrasound processing. The intensity of these peaks revealed that thermal-ultrasound treatment of corn starch with Arabic gum gave more potential to starch's microstructure to bound water. There were no changes observed in the chemical structure of corn starch-Arabic gum mixtures after ultrasound due to the addition and elimination of other peaks in FT-IR spectra. This phenomenon demonstrated the physical modification of thermal-ultrasound treatment of corn starch in the presence of Arabic gum. Moreover, compared with corn starch, Arabic gum increased the intensity of peaks in corn starch-Arabic gum mixture without treatment (S-G).

The crystallinity index of samples can be determined from the absorbance ratios of R1047/1022 and R1022/995 cm^−1^ ratios that are calculated in Table 3. These ratios indicate the short-range crystallinity associated with the double-helix packing enclosed by the inner granule microstructure. Generally, the higher R1047/1022 cm^−1^ ratio and the lower R1022/ 995 cm^−1^ absorbance ratio showed a higher relative crystallinity. These trends were observed in Table 3; but the microstructural characterization of treated starch was completed by XRD test.

### X-ray diffraction (XRD)

XRD patterns and relative crystallinity (%) of thermal-ultrasound-treated samples were reported in Fig. 2 and Table 3, respectively. The S and S-G samples showed an A-type X-ray pattern with four specific peaks at 2ϴ of 15.40, 17.30, 18.05, and 23.25. Moreover, thermal-ultrasound treatment at 25 and 45 °C temperatures scarcely altered the XRD patterns in comparison to S and S-G. These results considered that the crystallinity of samples remained unaffected. Only a slight reduction in the angle diffraction intensity of samples has been observed. Reductions in the degree of crystallinity were reported for oat starch after sonication^14^. This phenomenon was in accordance with the observation of SEM images of some granules that initiated to loss of their integrity. The relative crystallinity of treated samples at 65 °C decreased with increasing time and the lower value was 21.88% for S-G6560 (Table 3). In general, the relative crystallinity of S-G sample after sonication remained close to their corresponding native starch (S), and with an increase in sonication temperature, it showed a slight decrease, except the sample sonicated at 65 °C which was shown to be gelatinized; therefore, the crystallinity was very low.

### Pasting measurements

The pasting properties of the treated corn starch-Arabic gum samples are studied as a function of thermal heating. The rheological parameters are calculated from pasting curves and reported in Table 4. All samples showed significant differences in their pasting parameters. The Ts of samples sonicated at 65 °C for 30 and 60 min showed a significantly higher value in comparison to others. With increasing sonication temperature to 60 °C, the Tmax value changed significantly (*p* < 0.05) from 72.63 to 84.17 °C. Tmax increased from 73.22 to 75.51 °C with the addition of Arabic gum to corn starch. So, the Ts and Tmax increased with increasing temperature and time after treatment.

**Table 4:** Parameters obtained from the dynamic temperature sweep of thermal-ultrasound treated samples.

Sample	Ts (°C)	Tmax (°C)	$\upeta ^{*}_{{{\text{max}}}}$ (Pa s)	B (Pa s)	$\upeta ^{*}_{{\text{f}}}$ (Pa s)
S-G2530	66.86 ± 0.83^c^	75.00 ± 0.84^cde^	41.70 ± 6.79^b^	37.27 ± 6.25^b^	18.10 ± 3.53^c^
S-G2560	66.26 ± 0.01^c^	76.10 ± 2.40^bc^	48.05 ± 14.49^b^	43.23 ± 13.68^b^	19.65 ± 5.59^c^
S-G4530	66.85 ± 0.81^c^	72.63 ± 0.82^e^	105.55 ± 23.12^a^	96.60 ± 21.49^a^	43.05 ± 7.00^a^
S-G4560	67.73 ± 0.01^c^	77.83 ± 0.00^b^	36.08 ± 7.05^b^	32.04 ± 6.40^b^	14.90 ± 3.39^c^
S-G6530	69.73 ± 0.01^b^	77.25 ± 0.81^bc^	43.54 ± 4.14^b^	38.08 ± 3.75^b^	20.05 ± 1.63^c^
S-G6560	73.79 ± 0.80^a^	84.17 ± 0.81^a^	40.17 ± 7.88^b^	26.77 ± 5.20^b^	32.80 ± 5.37^b^
S-G	66.85 ± 0.81^c^	75.51 ± 0.02^bcd^	50.20 ± 4.89^b^	46.22 ± 4.80^b^	18.65 ± 1.20^c^
S	66.29 ± 1.62^c^	73.22 ± 0.01^de^	102.45 ± 12.09^a^	94.50 ± 11.81^a^	41.60 ± 4.38^a^

^a–e^For each column, similar code letters are not significantly different at *p* < 0.05.

The maximum viscosity ($\upeta ^{*}_{{{\text{max}}}}$) of corn starch significantly decreased with the addition of Arabic gum. This phenomenon is related to the substitution of Arabic gum (0.25% wt) for corn starch (4.75% wt.). Moreover, Arabic gum restricted swelling of the starch granules and limited the increase in viscosity during heating. In another study by Chen et al.^24^, Arabic gum was found to lower the peak viscosity and swelling power leading to a delay of retrogradation. These characteristics may be related to many parameters such as molecular structures, amylose and amylopectin interactions, and ionic charges of starch and hydrocolloids^24^. The maximum viscosity of treated samples increased from 25 to 45 °C and this value decreased from 45 to 65 °C again. S-G4530 samples had the highest value among others. Except for S-G4530, the B value of treated samples decreased after thermal-ultrasound treatment and showed the highest value in the S-G4530 and S-G6560 samples. The final viscosity ($\upeta ^{*}_{{\text{f}}}$) indicates the retrogradation characteristics of samples. The final viscosity of S-G decreased in comparison to S. Arabic gum can retard gelatinization and/or retrogradation. Interactions between hydrocolloid and amylose molecules that compete with amylose-amylose intermolecular interactions have been proposed as the reason for reduced retrogradation^25^. A good correlation was observed between leached amylose content and the retrogradation behaviors of samples. As the leached amylose content was lower, the final viscosity was also reduced after the thermal-ultrasound treatment. For example, the final viscosity of S-G45-30 was higher due to the high level of leached amylose. Generally, the final viscosity ($\upeta ^{*}_{{\text{f}}}$) of treated samples decreased after thermal-ultrasound treatment and then increased with increasing temperature and time. A decrease in the final viscosity of the treated sample indicated that retrogradation could be retarded under thermal-ultrasound treatment.

Based on the above results, thermal-ultrasound of corn starch in presence of Arabic gum caused to alter in the pasting properties of starch. In some cases, the pasting properties of corn starch-Arabic gum mixtures have been improved, for example, increasing the viscosity and decreasing the retrogradation characteristics.

## Materials and methods

### Materials

Corn starch (S4126) and Arabic gum were purchased from Sigma-Aldrich (St. Louis, MO, USA) and Daejung (Shiheung, South Korea), respectively. The manufacturer has reported corn starch's composition as follows: 11.3% wt. moisture content and pH 5.2. All other chemicals were of analytical grade. Moreover, all solutions were prepared with distilled water.

### Sample preparation and ultrasound treatment

The treatment of corn starch (S) was performed by ultrasound of starch with Arabic gum (G) combination. Firstly, Arabic gum (1.25 g) was slowly dissolved in distilled water (475 ml) and vigorous stirring with a magnetic stirrer. Then, corn starch (23.75 g) was added to the previous gum solution, and the solution was stirred continuously for 30 min at 23 ± 1 °C. The suspensions were treated in an ultrasound bath (Maxwell TECHNOLOGIES®, USA) at 30 kHz and a power of 270 W/cm^2^ based on different conditions. Different temperatures (25–65 °C) and time (30 min and 60 min) of sonication were selected based on starch pasting during heating. Aimed at controlling bath temperature, a thermocouple was employed and cooled down to an appropriate temperature by adding a little iced water. Each treatment was carried out based on 5% wt. total biopolymer concentration and 95:5 biopolymer ratio (S:G) at the center of the sonication bath. After treatment, all suspensions were freeze-dried. The control sample was not sonicated (with and without Arabic gum); but freeze-dried after dissolving in water. The treated samples are named as follows: S-G2530 = treated at 25 °C for 30 min; S-G2560 = treated at 25 °C for 60 min; S-G4530 = treated at 45 °C for 30 min; S-G4560 = treated at 45 °C for 60 min; S-G6530 = treated at 65 °C for 30 min; S-G6560 = treated at 65 °C for 60 min; S-G = corn starch- Arabic gum mixtures without treatment; S = corn starch without treatment.

### Amylose content

The amylose content of the S-G samples was determined by the method of Sodhi and Singh^26^. 20 mg of starch sample (based on starch weight) was taken and 10 ml KOH 0.5 N was added to it. The suspension was mixed vigorously. The dispersed sample was transferred to a 100 ml volumetric flask and distilled water was added to it. 10 ml of starch solution was pipetted to the 50 ml volumetric flask and 5 ml HCL 0.1 N and 0.5 ml iodine reagent were added. Distilled water was added to 50 ml and absorbance was measured at 625 nm. The amylose content of samples was calculated based on standard curves of amylose solutions.

### Syneresis (%)

S-G suspension (5% wt. concentration) was heated at 90 °C for 30 min in a water bath. After that, the sample was cooled to 4 °C and stored for 2- and 7-days storage at 4 °C. Syneresis was calculated based on the amount of released water (%) after centrifugation at 3200 × *g* for 15 min^26^.

### Paste clarity

Paste clarity of samples was determined at 4 °C after 2- and 7-days storage. Firstly, starch suspension (1% wt. concentration) was prepared and heated for 30 min at 95 °C. After cooling, the absorbance value of the starch paste was measured at 650 nm with a UV–visible spectrophotometer^27^.

### Swelling power and solubility

The swelling power and solubility of S-G starches were determined by the method of Okonkwo et al.^28^. Briefly, starch-gum samples were suspended in distilled water to obtain 1% wt. total concentration. The suspensions were heated at 25, 45, 65, and 85 for 30 min with continuous shaking. After rapid cooling to room temperature, the samples were centrifuged for 20 min (2400 g) and the weight of sediment was measured (m_2_). Then, the precipitated paste and supernatant were dried at 110 °C to a constant weight (m_3_ and m_4_, respectively). Swelling power (SP) and solubility (S) were calculated by Eqs. (1) and (2):

1 $${\text{Swelling power}}\left( {{\text{SP}}} \right) = \frac{{{\text{m}}_{{2}} }}{{{\text{m}}_{{3}} }} \times {1}00\quad \left( {{\text{Hydrated starch granules }}\left( {\text{g}} \right)/{\text{dry granules in precipitated paste }}\left( {\text{g}} \right)} \right)$$

2 $${\text{Solubility}}\left( {\text{S}} \right) = \frac{{{\text{m}}_{{4}} }}{{{\text{m}}_{{1}} }} \times {1}00\quad \left( {{\text{Soluble solid }}\left( {\text{g}} \right)/{\text{whole starch sample }}\left( {\text{g}} \right)} \right)$$

m_1_ is the weight of the initial dry sample.

### Differential scanning calorimetry (DSC)

Thermal properties of the freeze-dried samples were determined using a differential scanning calorimeter (SANAF DSC model, Iran). 5 mg starch sample was weighed in a hermetic aluminum pan and distilled water was added to it until the starch suspension reached 50% wt. concentration. The sealed pan was transferred to the heating equipment of the DSC and an empty pan was applied as a reference. All samples were scanned from 20 to 120 °C with a 10 °C/min heating rate. The onset (To), Peak (Tp), and gelatinization enthalpy (ΔH) were calculated from thermograms^11,29^.

### Scanning electron microscopy (SEM)

The microstructure of the freeze-dried samples was examined by scanning electron microscope (ZEISS, Model EVOLS10, Germany). A small amount of sample was fixed on the surface of the stub and dried at room temperature (23 °C) for one day. Then, all samples were coated with gold–palladium before observation. The image of the sample was taken at the range 2000 × and 3000 ×, and the applied voltage was 10 kV^30^.

### Fourier transform infrared (FT-IR) spectra

The infrared spectra of the freeze-dried samples were performed by an FT-IR spectrometer (FTIR 8400 s, Shimadzu, Japan). The wavenumber was applied in the range 400–4000 cm^−1^. All samples were mixed with KBr and the resolution was (4 cm^−1^)^2^.

### X-ray diffraction (XRD)

X-ray diffraction patterns of the freeze-dried samples were obtained with an X-ray diffractometer (Bruker, Model D8, Germany) by the following method of Yang et al.^31^ with slight modification. Diffraction was performed on an X-Ray Diffractometer with Cu-Ka radiation (λ = 0.154 nm) at 40 kV and 40 mA. Scanning was done at a reflection angle of 5° (2θ) to 70° (2θ) with a rate of 0.05°/min. Relative crystallinity (RC) was calculated as the ratio of the crystalline reflation area to the overall diffraction area between 2θ values of 5° and 40°.

### Pasting measurements

The rheological properties of samples were studied by a rheometer (Anton-Paar, Physica MCR 301, Austria) with the cone and plate measuring system (5 cm diameter, 2° angle, 206 µ gap size). The sample temperature was precisely (± 0.01 °C) controlled by a Peltier-plate system and a physical circulating thermostatic water bath. Heating and cooling rates were selected based on preliminary experiments.

Initially, starch suspensions were prepared by 5% wt. concentration. The viscoelastic properties of treated samples as a function of temperature were programmed at a strain of 0.5% and 1 Hz frequency. Aimed at preparing starch paste, sample suspensions were rested for 1 min at 50 °C, and then heated from 50 to 95 °C at a rate of 5 °C/min, held at 95 °C for 1 min, and cooled from 95 to 50 °C at a rate of 5 °C/min. Before beginning, the starch paste was held for one min at 50 °C and then initiated. Starch retrogradation may occur at this temperature. To prevent evaporation in samples at 95 °C, a thin layer of mineral oil was used around the edges of the measuring system probe. Based on mechanical spectra as a function of temperature, the below parameters were calculated from pasting curves: (1) T_S_: the temperature at which complex viscosity ($\upeta ^{*}_{{{\text{max}}}}$) starts to increase suddenly. (2) T_max_: the temperature related to maximum $\upeta ^{*}$. (3) $\upeta ^{*}_{{{\text{max}}}}$: the value of maximum $\upeta ^{*}$. (4) B: the difference between the maximum and minimum $\upeta ^{*}$. (5) the value of $\upeta ^{*}$ at end of the test (50 °C)^9,16^.

### Statistical analysis

The analytical determination was done by SAS 9.1 software version 2008 (https://www.sas.com/en_us/home.html). The mean of data was comprised of analysis of variance method (Proc GLM) at a significant level of 95% (*p* < 0.05). A least significant difference (LSD) was employed to determine the statistical differences among the mean values.

## Conclusion

This research studied the physicochemical, structural, morphological, and pasting properties of thermal-sonicated corn starch in presence of Arabic gum. Based on the results, amylose leakage, solubility, swelling power, syneresis of starch gels, paste clarity, and pasting characteristics were induced by thermal-ultrasound treatment in presence of Arabic gum. Moreover, the thermal properties and X-ray diffraction of corn starch changed after treatment. Scanning electron microscopy and FT-IR spectra of samples confirmed the modification of structural properties. Temperature and time are selected as key parameters in this modification method.

Therefore, ultrasound treatment could be a suitable method for the modification of starch granules. On the other hand, currently, thermal treatment due to the improved thermal stability and decreased extent of retrogradation is of great interest as a physical modification technique. Moreover, it is well known that the addition of hydrocolloids improved starch functionality by altering its rheological/ structural and gelatinization properties. Therefore, thermal-ultrasound treatment of corn starch and Arabic gum mixtures offer opportunities for the modification of starch and new biopolymers with application in the food industries based on the suitable changing of ultrasound conditions.

## Data Availability

The datasets used and/or analysed during the current study available from the corresponding author on reasonable request.
