# Author Correction: Modification of corn starch by thermal-ultrasound treatment in presence of Arabic gum

**DOI:** 10.1038/s41598-022-26356-y

**Published:** 2022-12-16

**Authors:** Abdolkhalegh Golkar, Jafar Mohammadzadeh Milani, Ali Motamedzadegan, Reza Esmaeilzadeh Kenari

**Affiliations:** Department of Food Science and Technology, Sari Agricultural Sciences and Natural Resources University, Sari, Iran

Correction to: *Scientific Reports* 10.1038/s41598-022-23836-z, published online 11 November 2022

The original version of this Article contained a typo in the Abstract.

“The relative crystallinity index of samples changed in the range 21.88-35.42–% and decreased with rising time and temperature.”

now reads:

“The relative crystallinity index of samples changed in the range 21.88–35.42% and decreased with rising time and temperature.”

The original Article has been corrected.

